# Mental health and work overload in informal caregivers: a mixed-method study

**DOI:** 10.47626/1679-4435-2025-1409

**Published:** 2025-06-05

**Authors:** Manoel Borges da Silva Junior, Manoela Gomes Reis Lopes, Erisonval Saraiva da Silva, Jardeliny Corrêa da Penha, Sandra Lorena Beltran Hurtado, Mychelangela de Assis Brito, José Wicto Pereira Borges

**Affiliations:** 1 Universidade Federal do Piauí, Teresina, PI, Brazil; 2 Universidade de São Paulo, São Paulo, SP, Brazil

**Keywords:** stroke, public health, informal caregivers, caregiver burden, acidente vascular cerebral, saúde pública, cuidadores informais, sobrecarga do cuidador

## Abstract

**Introduction:**

Stroke is one of the most common causes of sequelae and disabilities worldwide. The sequelae often restrict performance of daily activities, requiring the help of caregivers.

**Objectives:**

To assess the mental health and work overload of caregivers of stroke survivors based on ergonomics activity analysis.

**Methods:**

This mixed methods study included 76 informal caregivers, 11 of whom also participated in a qualitative study phase. The Self-Reported Questionnaire-20 was used to investigate mental distress, while the Zarit Burden Interview scale was used to assess work overload. The qualitative phase included an interview.

**Results:**

The prevalences of mental distress and work overload were 44.7% and 64.5%, respectively, and they were strongly correlated (r = 0.707, p < 0.001). Caregiver distress was associated with comorbidities (p = 0.003) and illiterate patients (p = 0.04). No variables were associated with work overload. Qualitative ergonomics analysis resulted in two categories: (1) description of the work routine and (2) the physical, mental, and emotional exhaustion of caregivers. The quantitative data converged into two categories: description of the work routine and its effects on the lives of caregivers.

**Conclusions:**

the results indicate the need for structural changes in social and health programs for caregivers of stroke survivors.

## Abstract (pt)

**Introdução:**

O acidente vascular cerebral é uma das causas mais recorrentes de sequelas e incapacidades no mundo. Essas sequelas frequentemente geram restrições na realização das atividades de vida diária, o que dificulta o retorno às atividades cotidianas.

**Objetivos:**

Avaliar a saúde mental e a sobrecarga de trabalho de cuidadores de sobreviventes de acidente vascular cerebral, com base na Ergonomia da Atividade.

**Métodos:**

Estudo de métodos mistos, realizado com 76 cuidadores informais, dos quais 11 também participaram da fase qualitativa do estudo. Para investigar o sofrimento mental, utilizou-se o Self Reported Questionnaire-20, e para a sobrecarga de trabalho, a escala Zarit Burden Interview. Já na etapa qualitativa, foi realizada uma entrevista mediante utilização de um roteiro que continha uma questão norteadora.

**Resultados:**

A prevalência do sofrimento mental foi de 44,7% e da sobrecarga de trabalho, de 64,5%, com forte correlação (r = 0,707; p < 0,001). O sofrimento mental apresentou associação com comorbidade (p = 0,003) e com o paciente não alfabetizado (p = 0,04). Não se observaram associações com a sobrecarga de trabalho. As análises qualitativas, sob a perspectiva da Ergonomia da Atividade, geraram duas categorias: descrição do trabalho habitual e deterioração e desgaste físico, mental e emocional dos cuidadores. Os dados quantitativos convergiram com as categorias referentes à descrição do trabalho habitual e aos efeitos na vida dos cuidadores.

**Conclusões:**

Os resultados indicam a necessidade de elaboração de propostas de mudanças estruturais em programas sociais e de saúde para essas pessoas.

## INTRODUCTION

Stroke, a public health problem, is one of the leading causes of sequelae and disabilities in the world.^1,2^ These sequelae often restrict movement, cognition, and decision-making capacity,^3^ making it difficult to return to daily activities and leading to dependence on care, which affects the survivor’s quality of life.^4^ Thus, after hospital discharge, a caregiver becomes a daily requirement for survivors, and these caregivers are usually informal,^5^ i.e., individuals with no professional training who provide assistance to an acquaintance or family member, such as a spouse, parent, or child.^6^

Caregivers play a fundamental role in meeting basic human needs and the rehabilitation process.^5^ Caring for stroke survivors can be stressful, involving work overload and affecting the caregiver’s mental health,^6^ in addition to causing emotional, social, and economic overload for the family.^7^ This is because caregiving for stroke survivors is almost always a full-time activity, resulting in a new lifestyle based on the patient’s needs, which should be understood as a job. Thus, these individuals are susceptible to depression and anxiety symptoms, difficulty sleeping, and reduced quality of life.^8^

Understanding this situation from a work perspective can clarify the work structure of caregivers and support health-promotion policies for them. The relationship between work overload and mental health in caregivers of stroke survivors can also be assessed from the perspective of activity ergonomics, which allows us to understand the actual work of caregivers, its variability, possible adjustments, and room for improvement.

Activity ergonomics studies the relationships between workers and their work environment, focusing on human activity, clarifying the relationship between work activity and the health-disease process.^9^ Given the complexity of the topic, mixed methods studies can expand knowledge of these phenomena, intervening factors, and the subjective structuring of daily life. Within this context, the present study used activity ergonomics to assess mental health and work overload among caregivers of stroke survivors.

## METHODS

This convergent mixed methods study collected both quantitative and qualitative data, equally emphasizing both sets. Since there is no dominant approach, isolated interpretations are made in both methods, and the results are analyzed and discussed.^10^ Stroke survivors and their informal caregivers were included. The study was conducted in northeastern Brazil in two cities in the states of Maranhão and Piauí that are separated by the Parnaíba River.

A total of 312 stroke survivors participated in the study, of whom 98 had sequelae and required a caregiver. Of these 98 caregivers, 22 were formally employed and were thus excluded. The other 76 were informal caregivers, all of whom participated in this study, resulting in a census sample.^11^

Caregivers, who were identified through community health agents associated with Basic Health Units in the two cities, were invited to participate in the study and, after they provided written informed consent, data were collected between May and July 2023 through questionnaires and semi-structured interviews.

In the quantitative stage of the study, socioeconomic, demographic, and health data were collected from the caregivers. Mental distress was investigated with the Self-Reported Questionnaire, which consists of items that measure somatic symptoms of depressive/anxious mood, decreased vital energy, and depressive thoughts. The cut-off point for suspected psychological distress was ≥ 7 affirmative answers.^12^ Work overload was investigated with the Zarit Burden Interview, which has been validated for use in Brazil and consists of 22 items. Scores range from 0 to 4. Work overload was classified by scores ≤ 21 points (no overload) and > 22 (overload).^13^

In the quantitative stage, data were also collected from stroke survivors, such as sociodemographic and economic data, as well as clinical information on the survivor’s health status.

The data were analyzed in IBM SPSS Statistics 28.0.1.1. In the bivariate analysis, Pearson’s chi-square test was used for dichotomous variables, and the odds ratio was calculated to estimate the strength of the associations. Pearson’s correlation was used for continuous variables. A 5% significance level was applied in all tests.

Sampling was intentional in the qualitative stage. Caregivers who expressed an interest in discussing the topic and who had participated in the quantitative stage were included. Data collection was interrupted when no new elements emerged to support the theory.^14^ Since this stage aimed to clarify the caregiver’s work routine, they were asked to describe their daily activities at home with the stroke survivor.

Using Bardin’s thematic categorical content analysis, 11 interviews were conducted, recorded, transcribed, and analyzed. This analysis is divided into three stages: pre-analysis; exploration of the material or coding; and the processing of results, inference, and interpretations.^15^ When exploring the material and processing the results, the data were analyzed according to the theoretical framework of activity ergonomics, either supporting, complementing, or explaining aspects of the quantitative results.^16^ The following thematic categories were determined through the analysis: the work routine of informal caregivers; and the informal caregivers’ physical, mental, and emotional exhaustion. To preserve the anonymity of the interviewees, they were identified by codes beginning with the letter P, followed by numerals.

The study was approved by the research ethics committee of the Universidade Federal do Piauí and was conducted in accordance with National Health Council principles (opinion 5.981.191).

## RESULTS

Regarding the profile of informal caregivers, the following characteristics were observed: 63.2% were between 20 and 59 years old, 76.3% were women, 90.8% were Black, 72.4% were illiterate, 97.4% were related to the stroke survivor, 53.9% had comorbidities, and 52.6% were taking medication. Regarding the patient profile, the following characteristics were observed: 94.5% were over 60 years of age, 76.8% were men, 58.3% were Black, 58.3% were without a partner, 88.2% were illiterate, 97.2% were unemployed, 60.8% had retired before the stroke, and the income of 95.9% was above minimum wage.

The prevalences of mental distress and work overload among caregivers were 44.7% and 64.5%, respectively. The mean Self-Reported Questionnaire score was 6.97 and the mean Zarit Burden Interview score was 31.42, with a strong correlation between them (r = 0.707; p < 0.0001). There was an association between mental distress and comorbidities. Caregivers with comorbidities were 2.3 times more likely to have mental distress than those without comorbidities. There was no association between work overload and the other variables (Table 1).

**Table 1:** Sociodemographic characterization and association analysis with SRQ-20 and Zarit Burden Interview scores of informal caregivers of stroke survivors, 2024 (n = 76)

	n	%	Mental suffering (SRQ-20)						Work overload (Zarit)					
			No		Yes		p-value^1^	OR (95%CI)^2^	No		Yes		p-value^1^	OR (95%CI)^2^
			n	%	n	%			n	%	n	%		
Age range (years)							0.237						0.050	
20-59	48	63.2	29	38.2	19	25.0		1.761 (0.687-4.515)	21	27.6	27	35.5		2.852 (0.980-8.295)
≥ 60	28	36.8	13	17.1	15	19.7		b	6	7.9	22	28.9		b
Sex							0.098						0.824	
Male	18	23.7	13	17.1	5	6.6		2.600 (0.821-8.234)	6	7.9	12	15.8		0.881 (0.288-2.691)
Female	58	76.3	29	38.2	29	38.2		b	21	27.6	37	48.7		b
Race							5.130							
Black	69	90.8	36	47.4	33	43.4		5.130 (0.410-64.209)	20	26.3	49	64.5		-
Other	7	9.2	6	7.9	1	1.3			7	9.2	0	0.0		b
Marital status							1.087						0.101	
With a partner	46	60.5	23	30.3	23	30.3		1.087 (0.326-3.631)	13	17.1	33	43.4		1.019 (0.210-4.958)
Without a partner	30	39.5	19	25.0	11	14.5		b	14	18.4	16	21.1		b
Education level							0.806						0.409	
Illiterate	55	72.4	29	38.2	26	34.2		0.806 (0.204-3.177)	18	23.7	37	48.7		0.287 (0.047-1.766)
Literate	21	27.6	13	17.1	8	10.5			9	11.8	12	15.8		
Religion													0.753	
Catholic	67	88.2	36	47.4	31	40.8	1.000	-	24	31.6	43	56.6		-
Evangelical	8	10.5	5	6.6	3	3.9	1.000	-	3	3.9	5	6.6		-
Other	1	1.3	1	1.3	0	0.0		b	0	0.0	1	1.3		b
Employment status							0.290						0.257	
Employed	23	30.3	11	14.5	12	15.8		2.861 (0.409-20.007)	6	7.9	17	22.4		31.244 (1.279-763.297)
Unemployed	53	69.7	31	40.8	22	28.9			21	27.6	32	42.1		b
Receives income from caregiving							0.895						0.665	
No	74	97.4	41	53.9	33	43.4		b	26	34.2	48	63.2		b
Yes	2	2.6	1	1.3	1	1.3		1.289 (0.030-56.317)	1	1.3	1	1.3		0.377 (0.008-18.578)
Resides at same address as survivor							0.184						0.950	
No	11	14.5	6	7.9	5	6.6		b	4	5.3	7	9.2		b
Yes	65	85.5	36	47.4	29	38.2		3.397(0.559-20.625)	23	30.3	42	55.3		1.361 (0.145-12.760)
Has other profession							0.724						0.733	
No	58	76.3	31	40.8	27	35.5		b	20	26.3	38	50.0		b
Yes	18	23.7	11	14.5	7	9.2		0.547(0.019-15.533)	7	9.2	11	14.5		1.389 (0.013-143.817)
Related to survivor							0.498						0.665	
No	2	2.6	1	1.3	1	1.3		b	1	1.3	1	1.3		b
Yes	74	97.4	41	53.9	33	43.4		0.217(0.003-18.023)	26	34.2	48	63.2		1.389 (0.013-143.817)
Develops work activity							0.716						0.626	
No	56	73.7	30	39.5	26	34.2		b	19	25.0	37	48.7		b
Yes	20	26.3	12	15.8	8	10.5		1.795(0.076-42.163)	8	10.5	12	15.8		4.883 (0.032-743.300)
Monthly individual income							0.469						0.705	
< 1x MW	36	47.4	20	26.3	16	21.1		b	12	15.8	24	31.6		b
≥ 1x MW	40	52.6	22	28.9	18	23.7		0.621(0.171-2.256)	15	19.7	25	32.9		0.169 (0.025-1.152)
Comorbidities							0.002						0.086	
No	35	46.1	26	34.2	9	11.8		b	16	21.1	19	25.0		b
Yes	41	53.9	16	21.1	25	32.9		2.371(1.283-4.382)	11	14.5	30	39.5		1.348 (0.944-1.924)
Medication use							0.058						0.123	
No	36	47.4	24	31.6	12	15.8		b	16	21.1	20	26.3		b
Yes	40	52.6	18	23.7	22	28.9		1.650 (0.961-2.832)	11	14.5	29	38.2		1.305 (0.921-1.850

OR = odds ratio; MW = Brazilian federal minimum wage; SRQ-20 = Self-Reported Questionnaire.

* Chi-square test, with Yates correction, at the 5% level^1^.

† Odds ratio with 95%CI^2^.

Table 2 presents an association analysis between caregiver test scores and patient demographics. Patient illiteracy was associated with mental suffering in caregivers (p = 0.04). No associations were observed between patient variables and work overload among caregivers.

**Table 2:** Sociodemographic and economic characterization of stroke survivors and association analysis with SRQ-20 and Zarit Burden Interview scores of their informal caregivers, 2024 (n = 76)

Survivor variables	n	%	Caregiver variables											
			Mental suffering (SRQ-20)						Work overload (Zarit)					
			No		Yes		P-value^1^	OR (95%CI)^2^	No		Yes		P-value^1^	OR (95%CI)^2^
			n	%	n	%			n	%	n	%		
Age range (years)							0.211						0.651	
< 60	4	5.55	1	2.4	3	8.8		-	1	3.7	3	6.1		-
≥ 60	72	94.5	41	97.6	31	91.2		b	26	96.3	46	93.9		b
Sex							0.338						0.233	
Male	43	76.8	22	52.4	21	61.8		1.664 (0.587-4.714)	13	48.1	30	61.2		1.857 (0.672-5.134)
Female	33	23.2	20	47.6	13	38.2		b	14	51.9	19	38.8		b
Race							0.164						0.985	
Black	48	58.3	30	71.4	18	52.9		0.474 (0.166-1.355)	18	66.7	30	61.2		0.990 (0.345-2.842)
Other	28	41.7	12	28.6	16	47.1		b	9	33.3	19	38.8		b
Marital status							0.654						0.590	
With partner	28	41.7	17	40.5	11	32.4		0.781 (0.264-2.310)	11	40.7	17	34.7		0.745 (0.256-2.170)
Without a partner	48	58.3	25	59.5	23	67.6		b	16	59.3	32	65.3		b
Education level							0.044						0.540	
Illiterate	68	88.2	41	97.6	27	79.4		0.079 (0.007-0.937)	25	92.6	43	87.8		0.562 (0.089-3.547)
Literate	8	11.8	1	2.4	7	20.6		b	2	7.4	6	12.2		b
Employment status							0.429						0.794	
Employed	2	2.8	1	2.4	1	2.9		4.547 (0.107 193.458)	1	3.7	1	2.0		0.624 (0.018-21.385)
Unemployed	74	97.2	41	97.6	33	97.1		b	26	96.3	48	98.0		b
Retirement/benefits														
Yes, after stroke	23	36.4	13	31.0	10	29.4	0.711	0,840 (0,259-2,722)	7	25.9	16	32.7	0.629	1.314 (0.435-3.970)
No	2	2.8	1	2.4	1	2.9	1.000	364169278.261 (0.000-)	1	3.7	1	2.0	1.000	-
Yes, before stroke	51	60.8	28	66.7	23	67.6		b	19	70.4	32	65.3		b
Income (MW)							1.000						1.000	
< 1	3	4.1	2	4.8	1	2.9		-	1	3.7	2	4.1		-
≥ 1	73	95.9	40	95.2	33	97.1		b	26	96.3	47	95.9		b

OR = odds ratio; MW = Brazilian federal minimum wage; SRQ-20 = Self-Reported Questionnaire.

* Wald test, 5% level^1^.

† OR, with 95%CI^2^.

Analysis of the qualitative data led to seven units of analysis that resulted in five themes, which were structured into two categories: description of the caregiver’s work routine and the physical, mental, and emotional exhaustion of caregivers. Chart 1 presents these thematic categories.

**Chart 1:** Themes and categories derived from interviews with informal caregivers

Examples of recording units	Units of analysis	Themes	Categories
*“I wake up early to make breakfast, take care of things like cleaning, serving breakfast, administering medications and leaving everything in order.”* [P01]	Domestic activities/direct care	Being a caregiver vs. household chores	Description of the caregiver’s work routine: a double workload
*“After serving breakfast, I clean the house, make food […], I live like a slave.”* [P02]	Domestic activities/direct care		
*“It’s always me in this house. I take care of him, make breakfast, wash clothes, clean the house, do everything. I feel tired, stressed; I can’t take it anymore.”* [P09]	Domestic activities/direct care		
*“I wake up early to take care of my dependents and do household chores. […]*” [P10]	Domestic activities/direct care		
*“I give him a bath, and move him back to his wheelchair, […] Then I do all the housework, like cleaning the house, the bathroom […]*” [P04]	Domestic activities/direct care		
*“After giving him breakfast, I have to clean the house, make food […], in addition to bathing and feeding him, I do all of that alone.”* [P11]	Domestic activities/direct care		
*“In addition to my caregiving activities, I have a small shop to supplement my family income. At noon, I bathe him, feed him, and organize the house […] and then I go back to the shop again.”* [P07]	Work activities/direct care	Being a caregiver vs. other work activities	
*“Most of the time I’m alone. It’s very difficult, but it’s a job I have to do; he’s my father.”* [P01]	Emotional overload	Emotional distress of solitary care	Physical, mental and emotional exhaustion of caregivers
*“I take care of him all by myself […] everything is difficult. I feel like giving up on everything, but who could I leave my father with?”* [P08]	Emotional overload		
*“There are days when I think I’m going to run down the street naked because I’m so stressed. It’s a headache, it’s stress […]”* [P01]	Mental overload	Mental exhaustion in the daily life of a caregiver	
*“I’m already stressed out with all of this; taking care of the house isn’t easy. Imagine what it’s like for a sick person who can’t walk. I’ve been having to do everything alone for almost 3 years.”* [P02]	Mental overload		
*“I live exhausted, I live with stress. I feel angry sometimes, so for me this is no good.”* [P03]	Mental overload		
*“It’s difficult to take care of him because I can’t handle it, so for me it’s difficult, it’s suffering.”* [P04]	Mental overload		
*“I can’t handle doing very much anymore […], but getting him to pee, to bathe him, is very difficult, stressful.”* [P05]	Mental overload		
*“…nobody helps me. I don’t want this life anymore […]”* [P10]	Mental overload		
*“Because she’s heavy I sometimes feel it here in my lower back.”* [P06]	Physical symptoms	Pain and physical exhaustion in the daily life of a caregiver	
*“I’m overwhelmed by everything. I get tired, I have a headache, I need someone to help me take care of myself.”* [P08]	Physical symptoms		
*“She’s too heavy, she can’t do anything on her own, I need help […]”* [P10]	Physical symptoms		

The first thematic category, the caregiver’s work routine, included statements describing the caregivers’ activities and exhausting workload. *“I wake up early, make breakfast. Then I get him out of bed, clean him, serve him breakfast, give him his medicine, then wash the dishes, make lunch, clean the house, and wash clothes.”* [P01]

*“When I get up, I go to the kitchen to make breakfast for the hungry people to eat. Here, I make breakfast for these people to eat, then I wake them up to give them their food. But before that, if they have peed themselves during the night, we have to change their clothes so they don’t stink. I make lunch, wash clothes, clean the house, and clean their room. I don’t stop working, except when I go to sleep.”* [P10]

*“When I bathe him, I transfer him from the wheelchair to the bath chair. This part is very difficult and I have to be careful not to lose my grip, because he has fallen at this point before. My arms hurt and I lose strength because I am old and I don’t have the strength. I suffer a lot because I can’t handle it, but no one notices. When it’s time to put him to bed and take him out of bed, it’s a pain, because the bed he sleeps in is too high for me. I can’t do it because he is stiff, I don’t have enough strength and he doesn’t help at all. All of this takes longer during feeding because of the difficulty he has eating, and this demands more work from me, making me more stressed, more tired…”* [P04]

*“I have a small shop because welfare benefits don’t cover everything. Sometimes I have to go out and run errands and my wife stays here watching the shop. Then I come back and look after at the shop. I always close at noon (sometimes earlier) because I need to bathe him, feed him, he can sit up alone and I feed him.”* [P07]

*“I take care of him by myself. When I need to go somewhere, one of them stays here. Everything is difficult. I feel like giving up on everything, but who could I leave my father with?”* [P08]

*“I need help, no one helps me. This is the hardest part for me, I’m exhausted.”* [P10]

*“I do everything by myself. I can’t handle it. I do it because there’s no one else to do it. I’m tired, worn out, and when it’s time to clean or change a diaper, I feel pain all over my body, I go crazy, it makes me feel anxious because it’s all on me.”* [P11]

**Figure 1:**
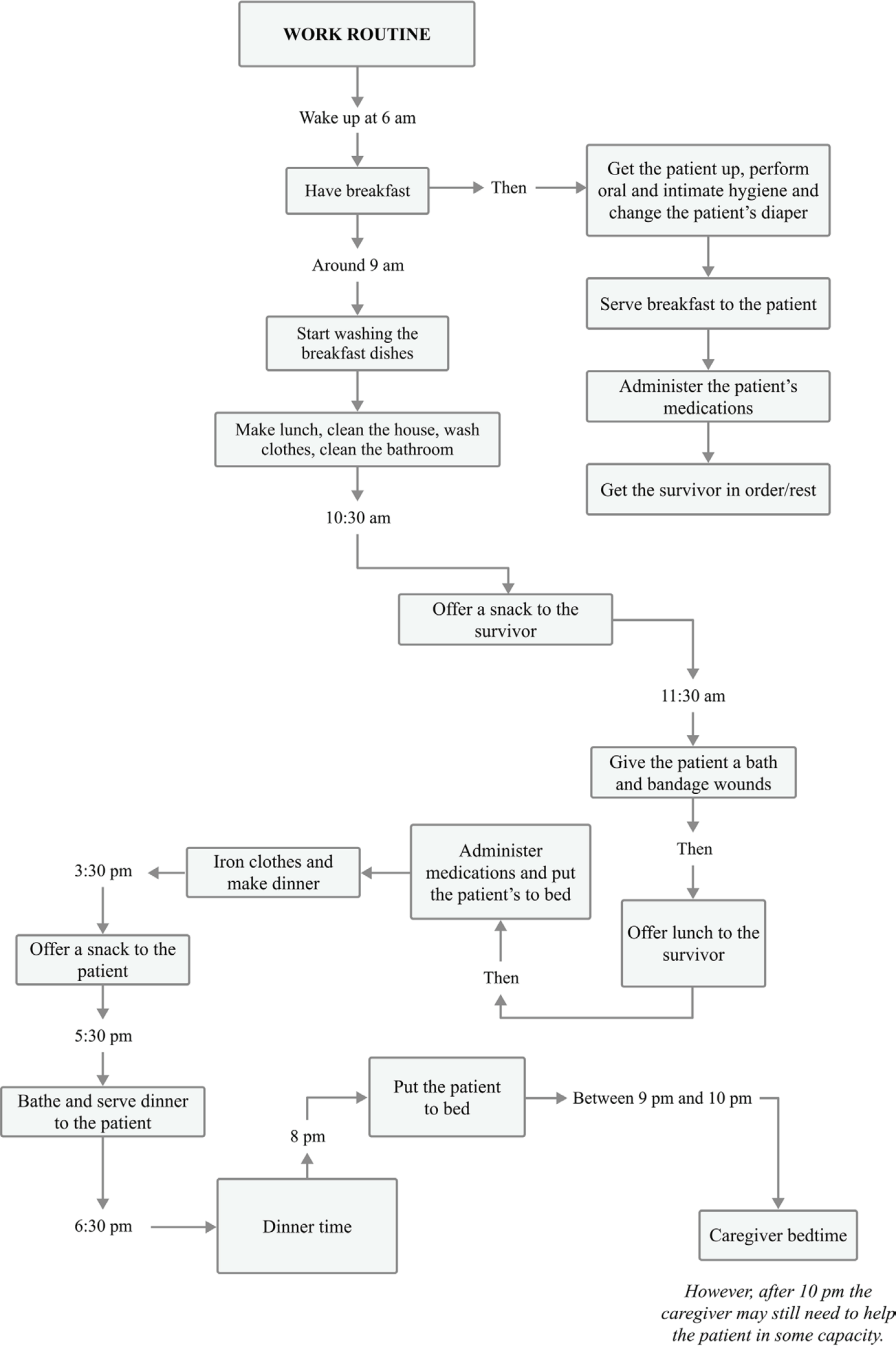
Flowchart of the informal caregiver’s daily activities.

Figure 1 presents a more detailed description of the findings of the first thematic category, detailing the caregivers’ activities, which showed a repetitive daily routine with a double workload (domestic and care work). They begin their activities at 6 am and finish at 10 pm, averaging 16 hours a day with practically no breaks.

In the second category, the physical, mental, and emotional exhaustion of caregivers, their statements revealed a joyless life without time for self-care. Fatigue and stress are prevalent in the daily lives of caregivers, which highlights their frailty. They require care because they have no quality of life at work and do not have a healthy, peaceful environment in which to carry out their activities.

*“Because there are days when I think I’m going to run down the street naked due to stress. It’s a headache, it’s stress, it’s having to take care of the boy, the husband, the dad, the house, and cook. I get tired, it’s terrible for me, my mind isn’t in good shape.”* [P01]

*“Because I’m already stressed. Taking care of the house and an ill person is very stressful, I’ve been living in this agony for 3 years. The other children don’t help, only my husband, and it ends up getting left up to the old slave here.”* [P02]

*“I’m always tired, I’m always stressed, I get migraines all the time. I get angry sometimes, so to me this is not good.”* [P03]

*“I don’t have a life anymore. I stay locked up inside the house. I don’t leave the house, my life has changed for the worse […]. It’s horrible. I’m going crazy, because my life is hell. People who live in a situation like this have no happiness. Before, I had joy in life, but nowadays I think death would be better than this.”* [P04]

*“I can’t do much anymore, but I still feel young for my age, because there are people my age who can’t handle it. But getting him up to pee, to bathe him is very difficult, because my arms always hurt from doing it.”* [P05]

*“Since she’s a bit heavy, I sometimes feel it here in my lower back.”* [P06]

*“This overload makes me tired, gives me a headache, I need someone to help with the caregiving.”* [P08]

*“She’s too heavy; she can’t do anything on her own. I’m tired, I need help, no one helps me. I don’t want this life anymore. I’m getting sick. I’m afraid of dying of a heart attack like we see on TV all the time, where someone died of a heart attack, fell down and died.”* [P10]

## DISCUSSION

In light of the above, the combination of patient care with daily activities, such as cleaning the house, caring for children, and cooking should be considered a double workload that requires a number of skills. This can become exhausting, having physical effects, producing stress, compromising health and, consequently, quality of life.^17,18^

Another aspect emphasized in the participants’ statements was how their lives changed after becoming caregivers. The sudden changes in social life and restrictions in leisure activities, such as attending worship services or parties, were clearly detailed in their statements. Their lives became exclusively dedicated to caregiving, a life without joy, support, or time left for self-care.

Through the daily, repeated performance of care activities for the dependent patient, family caregivers neglect self-care. This could explain why most caregivers in this study were overloaded had lower confidence regarding their own health, which is associated with immune dysregulation, coronary artery disease, and increased mortality.^19,20^

Furthermore, the lack of support for informal family caregivers directly highlights the challenges of self-care, which can result in stress, anxiety, and physical and psychological problems.^19^ An exploratory study observed that a lack of support has a considerable impact on the lives of caregivers, favoring overload and illness.^21,22^ Thus, in addition to the high workload, informal caregivers have little time to care for themselves, which increases their burden. Most caregivers dedicate themselves to caring for their dependent family member practically 24 hours a day, which limits their time for rest and recovery. This highlights the urgent need to improve support structures for caregivers,^23^ increasing rest time and minimizing their emotional and mental overload.

This study highlighted the vulnerable state of caregiver mental health, identifying suicidal, depressive, and stressful thoughts. Caregivers with comorbidities were two to three times more likely to suffer from mental distress. This corroborates a quantitative cross-sectional study of caregivers of advanced age in São Paulo, which found that those with depressive symptoms, pain, cognitive impairment, stress, and overload also had greater mental distress.^24^

We found that a small portion of caregivers had suicidal ideation, which coincides with a cross-sectional study on primary informal caregivers of people with stroke sequelae in João Pessoa, Brazil. A total of 23.2% of the participants in that study reported having suicidal thoughts once or twice a week.^25^ Suicidal ideation is often associated with constant suffering, psychological changes, and depression, which make the individual more vulnerable to emotional suffering and to thoughts about frustration and uselessness.^26^

One of the most common psychological symptoms among informal caregivers of people who require long-term care is depression,^22^ which can predispose caregivers to self-inflicted violence and suicide.^26^ A Turkish study on home caregivers found that 61.1% are depressed due to the stressful demands of their work and the lack of time for themselves.^27^

Furthermore, chronic stress can be manifested in informal caregivers through physical and psychological problems, which can worsen due to emotional overload, the effects on the caregiver’s personal life, and the care demands of the stroke survivor.^23^ Due to chronic stress, greater deficits in attention, working memory, and executive function have been found in caregivers of disabled older adults than in non-caregivers.^28^ Studies have observed worse mental health outcomes and increased levels of discomfort, anxiety, fatigue, and factors related to overload among caregivers. This is because bedridden older adults require greater care, which directly affects the dynamics of daily life, including the physical and mental health of caregivers.^29^

In addition to emotional and mental overload, our sample of caregivers reported difficulties performing tasks that required strength, such as moving patients, transferring them from the bed to a wheelchair, or changing their diapers, resulting in physical exhaustion and pain complaints. To deal with these difficulties, they relied on the help of others whenever possible for tasks such as bathing. Thus, physical suffering due to the high workload was apparent.

Because of the high workload, occupational factors in informal caregiving are associated with harmful effects, resulting in health problems that can go beyond physical and mental aspects, such as the exhaustion reported in most studies on the subject. Informal caregivers exposed to this high workload should receive support from the health system and help from other family members in caring for the patient.^30^

In view of the above, it is reaffirmed that prolonged caregiving can result in progressive exhaustion and an inability to provide self-care. The health of informal caregivers is a complex phenomenon and is associated with different factors that involve both the care demand and the clinical and therapeutic status of the patient. Therefore, primary health care assistance is essential for these caregivers.^22^ Primary health care is a basic and essential means of ensuring support and guidance for caregivers, both for the care they provide and their own health. Therefore, an interdisciplinary multidimensional assessment of caregivers and network assistance initiatives are needed to guarantee individualized support, incorporating assessment methods and interventions into the care process.^29^

## CONCLUSIONS

Most of caregivers in our sample presented high levels of depression, anxiety, and stress, as well as suicidal ideation. The correlations between the variables showed that caregivers with comorbidities have a higher risk of mental suffering than those without comorbidities. Slight work overload was detected in most caregivers, and a small portion had severe work overload. The needs of this population should be recognized and addressed, providing greater awareness among health care professionals about the importance of developing strategies to help informal caregivers perform their activities.

The study is limited by its small sample, which did not allow for an in-depth analysis of other causes of mental distress and work overload among caregivers. Nevertheless, it offers a valid contribution by assessing mental distress and work overload in informal caregivers, correlating data on significant variables in a population that tends to be neglected by the health sector.
